# Supplementary material for: In silico typing maps the natural diversity of Escherichia coli transporter-dependent capsules
Source: Nat Microbiol. 2026 Apr 23;11(5):1217–32. doi: 10.1038/s41564-026-02323-5 (PMC13171606; doi:10.1038/s41564-026-02323-5)
Supplement: Supplementary file 1 — Supplementary Figs. 1 and 2 and Legends of Supplementary Tables 1–19. [file 41564_2026_2323_MOESM1_ESM.pdf]

nature microbiology

Supplementary information

https://doi.org/10.1038/s41564-026-02323-5

In silico typing maps the natural diversity of *Escherichia coli* transporter-dependent capsules

In the format provided by the authors and unedited

Supplementary Information

Index

Supplementary Information
- Index
- Extended Data Figures
- Supplementary Figures
- Supplementary Tables
- References

Extended Data Figures

Extended Data Figure 1. Closely related capsule gene clusters identified in different K serotype reference strains do not support robust genetic differentiation of the respective serotypes, related to Figure 2. Capsule gene clusters were compared and rendered using clinker. The proportion of identical amino acids is displayed numerically and in grey-scale between opposing ORFs. The reported base identities are from global Needle-Wunsch alignments and the protein alignments were made with the Smith-Waterman algorithm. Gene assignments and functional predictions were made as described in Methods. Polysaccharides are depicted according to the conventions of the Symbol Nomenclature for Graphical Representation of Glycans and references for the displayed polysaccharide structures are provided in Supplementary Table 1. **A.** K2a and K2ab differ by acetylation of galactose in the repeat unit, consistent with a frameshift mutation in the putative acetyltransferase ORF in K2a. **B.** The K13 and K23 polysaccharides differ by acetylation of the Kdo residue in the repeat unit and the candidate acetyltransferases differ by two missense mutations. Although the observed mutations may explain the K2a/K2ab and K13/K23 serotypes, they do not provide a robust basis for genetically distinguishing these serotypes. Therefore, these serotypes are grouped under the names K2 and K13_K23, respectively, in our catalog (Supplementary Table 2). **C.** The K18 polysaccharide is an acetylated version of the K22 backbone, but the capsule gene clusters are identical (100% identity), and there is no candidate acetyltransferase gene within the clusters. The serological distinction between K18a and K18ab is unclear because chemical analysis of the polysaccharides revealed identical repeat unit structures. As there is currently no genetic basis for distinguishing these serotypes, they are grouped under the assignment K18_K22 in our catalog (Supplementary Table 2). **D.** Our analysis of the K54 and K96 gene clusters is consistent with earlier reports suggesting that the gene(s) responsible for threonine modification of the polysaccharide backbone are located outside of the *kps* locus. Pending identification of these gene(s), the K54 and K96 clusters are grouped under the assignment K96 in the current version of the catalog (Supplementary Table 2).

Extended Data Figure 2. Mass spectrometry clarifies K antigen structures, related to Figure 2. A. Capsule gene clusters from K serotype reference strains were compared and rendered with clinker, then colored according to gene assignments and predicted domain functions. Amino acid identity > 30% is displayed in greyscale between opposing genes. **B.** Published polysaccharide repeat unit structures for each K serotype (see references in Supplementary Table 1). **C.** Deconvoluted MALDI-TOF-TOF spectra of partially hydrolyzed and permethylated capsular polysaccharide (Methods). Peak labels indicate the assigned structure and corresponding expected m/z species calculated in GlycoWorkbench 2[92]. We note that O-acetyl groups are expected to be lost during the permethylation procedure and were not considered when assigning peaks.

Extended Data Figure 3. Evaluation of kTYPr on the RefSeq source data set. A. Upset plot of kTYPr results on 37,723 *E. coli* genomes from RefSeq, showing the distribution of *E. coli* genomes with complete and incomplete capsule biosynthesis loci. **B.** Absolute counts of K-types identified within the same RefSeq data set (n=37,723).

Extended Data Figure 4. Global diversity of K-types. A. Fraction of *kps*-positive genomes (defined as containing a complete *kps* locus, Methods) across geography. **B.** K-type proportion in hosts and environments. K-types with frequency < 5% are annotated as "other". **C.** K-type proportion in individual hosts and environments. K-types and sample origin are clustered according to Pearson's correlation. Host-environment combinations with < 15 occurrences were not considered. K-type groups are color-coded as in Fig. 3C. Hosts are color-coded according to their lower resolution groups shown in Fig. 3B. **D.** Proportion and absolute counts of K-types, color-coded according to groups as in Fig. 3C. K-types with proportion < 0.5% were annotated as "other". Only *kps*-positive genomes were considered. E. K-type proportion across geographic regions, grouped by hosts and environments. K-types with frequency < 8% are annotated as "other". Only *kps*-positive genomes were considered.

Extended Data Figure 5. K-type associations with phylogroups and human health and disease. A. Proportion of capsule groups across phylogroups in *E. coli* genomes from all hosts and environments. Groups are color-coded as in Fig. 3C, with grey indicating *kps*-negative genomes. **B.** Source of *E. coli* human isolates in the collection, grouped by phylogroup (n=11,556). 20 genomes to which no phylogroup could be assigned were removed. **C.** Fraction of *kps*-positive genomes (defined as containing a complete *kps* locus, Methods) in *E. coli* human gut metagenomes (Supplementary Table 13). **D.** Capsule group proportion in each phylogroup in asymptomatic carriage (A, corresponding to 2,762 *E. coli* MAGs from healthy individuals, Supplementary Table 13) and invasive *E. coli*-associated disease (I, 1,118 genomes of isolates from blood or cerebrospinal fluid from NCBI and 260 from blood from a published study on urosepsis), Methods. ns $p > 0.05$; * $p \leq 0.05$; ** $p \leq 0.01$; *** $p \leq 0.001$ (two-sided Fisher's exact test, Bonferroni correction). Groups are color-coded as in Fig. 3C, with grey indicating *kps*-negative genomes. **E-G.** K-type diversity (expressed as Simpson index, **E**), richness (**F**) and evenness (**G**) of phylogroups. The same data as in D were considered, filtering out phylogroups with ≤ 25 genomes in each group (asymptomatic, A or invasive, I). The number of *kps*-positive genomes in the two groups is indicated above each boxplot. Box limits correspond to first and third quartiles, with the median marked, and whiskers extending to the most extreme data points up to 1.5 times the interquartile range (IQR). **H.** K-type proportion across health groups defined as in Fig. 4B, considering all (left) or only *kps*-positive genomes (right). K-types with proportion < 2% (left) or < 4% (right) are grouped as "other".

Extended Data Figure 6. K-type associations with O and H antigens in human-associated *E. coli* genomes. A. Proportion of *kps*-positive genomes (top) and the number of unique K-types (bottom) per O-type. H-type composition is indicated for each O-type (bottom). Only O-types with > 25 genomes are shown. H-types with proportion < 30% are grouped as "Other". Genomes where no O-type-encoding locus was detected, and no O-type could therefore be assigned (Methods), are shown as "-". The same data as in Fig. 4D was considered. **B.** O-type proportion for each K-type. K- and O-negative genomes are not considered (n=4,699). K-type groups are color-coded as in Fig. 3C. O-type sugar composition was obtained from ECODAB[2,88]. Only unique sugars are listed. K12 co-occurrence with rhamnose-containing O-types is highlighted.

Supplementary Figures

Supplementary Figure 1. Kaptive analysis of the kTYPr catalog. The 85 *E. coli* transporter-dependent capsule gene clusters in the kTYPr catalog were profiled using Kaptive v.3.1.0[1] and EC-K-typing DB (v6.11.2025)[2]. The reference clusters are displayed as in Fig. 2, next to the corresponding Kaptive result (Supplementary Table 10), including the identified KL (Locus), assigned phenotype (Type), confidence (Typeable/Untypeable), percentage identity (%ID), and coverage (%COV). Established K antigen serotypes (K1-K103) that are not correctly assigned in EC-K-typing DB or are assigned to the wrong *kps* locus are highlighted in blue. KL assignments that are not equivalent (see Supplementary Table 7 and 8) to the displayed *kps* locus are highlighted in red. Results for capsule groups 2A, 2B and 3 are shown in panels A, B, and C, respectively. Pairwise nucleotide and amino acid sequence identities between all K-type ORFs and proteins are reported in Supplementary Table 6.

A

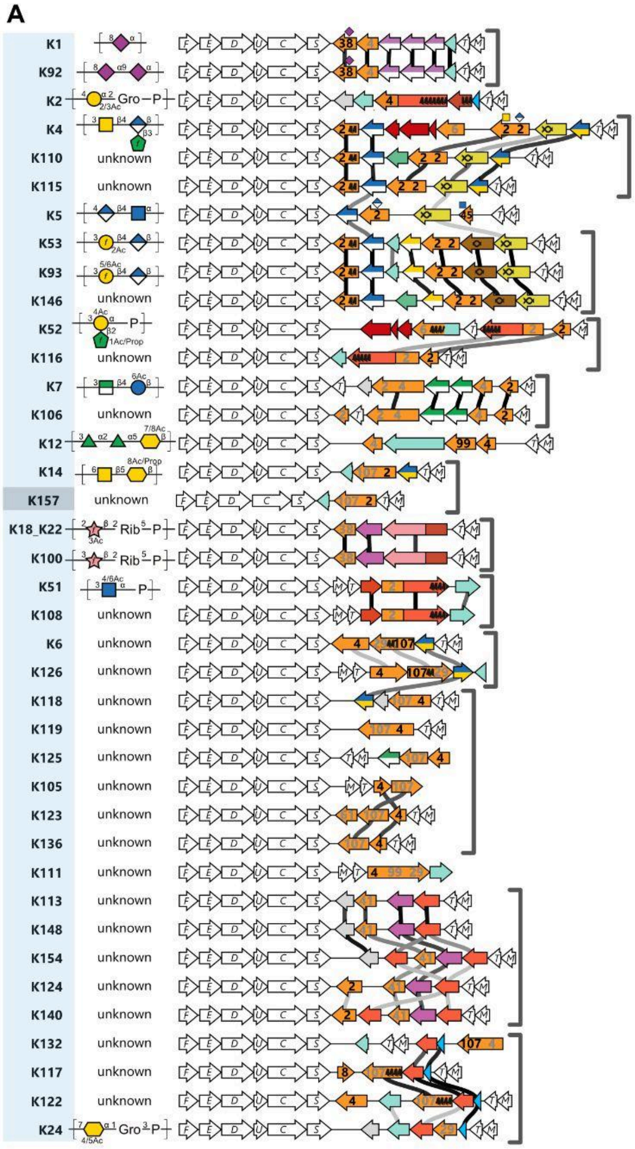

	Locus (Type)	Confidence (%ID, %COV)
K1	KL1 (K1)	Typeable (99.7%, 100.0%)
K92	KL92 (K92)	Typeable (100.0%, 100.0%)
K2	KL2 (K2)	Typeable (99.3%, 100.0%)
K4	KL4 (K4)	Typeable (98.6%, 100.0%)
K110	KL111 (KL111)	Typeable (99.8%, 99.8%)
K115	KL131 (KL131)	Typeable (99.4%, 100.0%)
K5	KL5 (K5)	Typeable (98.9%, 101.8%)
K53	KL53 (K53)	Typeable (99.4%, 100.0%)
K93	KL93 (K93)	Typeable (99.1%, 100.0%)
K146	KL93 (K93)	Typeable (96.7%, 96.1%)
K52	KL52 (K52)	Typeable (100.0%, 100.0%)
K116	KL123 (KL123)	Typeable (99.6%, 100.0%)
K7	KL7 (K7)	Typeable (99.5%, 100.0%)
K106	KL146 (KL146)	Typeable (99.6%, 100.0%)
K12	KL12 (K12)	Typeable (99.6%, 100.0%)
K14	KL14 (K14)	Typeable (98.6%, 99.9%)
K157	KL19 (K19)	Typeable (99.8%, 47.7%)
K18_K22	KL100 (K100)	Typeable (96.4%, 99.8%)
K100	KL100 (K100)	Typeable (100.0%, 100.0%)
K51	KL169 (KL169)	Typeable (99.9%, 100.0%)
K108	KL167 (KL167)	Typeable (100.0%, 99.9%)
K6	KL124 (KL124)	Typeable (99.9%, 100.0%)
K126	KL142 (KL142)	Typeable (99.4%, 100.0%)
K118	KL82 (K82)	Typeable (99.9%, 100.0%)
K119	KL119 (KL119)	Typeable (99.0%, 100.0%)
K125	KL137 (KL137)	Typeable (100.0%, 100.0%)
K105	KL129 (KL129)	Typeable (93.7%, 99.3%)
K123	KL135 (KL135)	Typeable (99.9%, 100.0%)
K136	KL129 (KL129)	Typeable (99.3%, 100.0%)
K111	KL117 (KL117)	Typeable (99.9%, 100.0%)
K113	KL118 (KL118)	Typeable (99.0%, 100.0%)
K148	KL118 (KL118)	Typeable (94.7%, 99.1%)
K154	KL154 (KL154)	Typeable (99.1%, 99.6%)
K124	KL125 (KL125)	Typeable (99.1%, 100.0%)
K140	KL161 (KL161)	Typeable (100.0%, 100.0%)
K132	KL144 (KL144)	Typeable (100.0%, 98.7%)
K117	KL122 (KL122)	Typeable (99.5%, 100.0%)
K122	KL126 (KL126)	Typeable (99.5%, 100.0%)
K24	KL24 (K24)	Typeable (99.5%, 100.0%)

Supplementary Figure 1

B

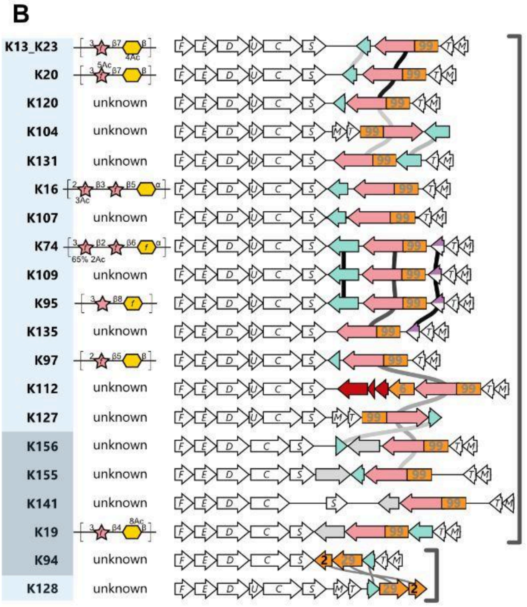

Locus (Type)	Confidence (%ID, %COV)
KL13 (K13)	Typeable (99.0%, 100.0%)
KL20 (K20)	Typeable (99.4%, 100.0%)
KL139 (KL139)	Typeable (100.0%, 100.0%)
KL112 (KL112)	Typeable (99.2%, 100.0%)
KL133 (KL133)	Typeable (100.0%, 100.0%)
KL16 (K16)	Typeable (99.3%, 100.0%)
KL120 (KL120)	Typeable (99.3%, 100.0%)
KL113 (KL113)	Typeable (99.6%, 100.0%)
KL113 (KL113)	Typeable (97.8%, 100.0%)
KL113 (KL113)	Typeable (97.0%, 100.0%)
KL141 (KL141)	Typeable (99.0%, 100.0%)
KL116 (KL116)	Typeable (99.9%, 100.0%)
KL115 (KL115)	Typeable (99.1%, 100.0%)
KL134 (KL134)	Typeable (99.9%, 100.0%)
KL19 (K19)	Typeable (99.5%, 47.7%)
KL19 (K19)	Typeable (99.5%, 47.7%)
KL19 (K19)	Typeable (99.4%, 47.8%)
KL19 (K19)	Typeable (100.0%, 100.0%)
KL19 (K19)	Typeable (96.7%, 53.0%)
KL117 (KL117)	Typeable (98.5%, 67.3%)

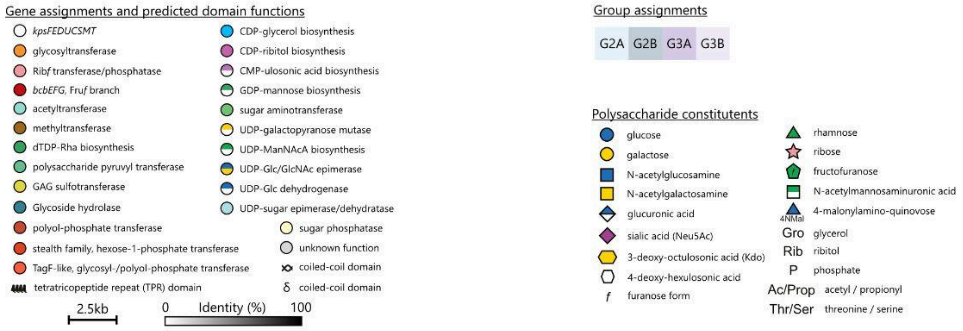

Supplementary Figure 1

C

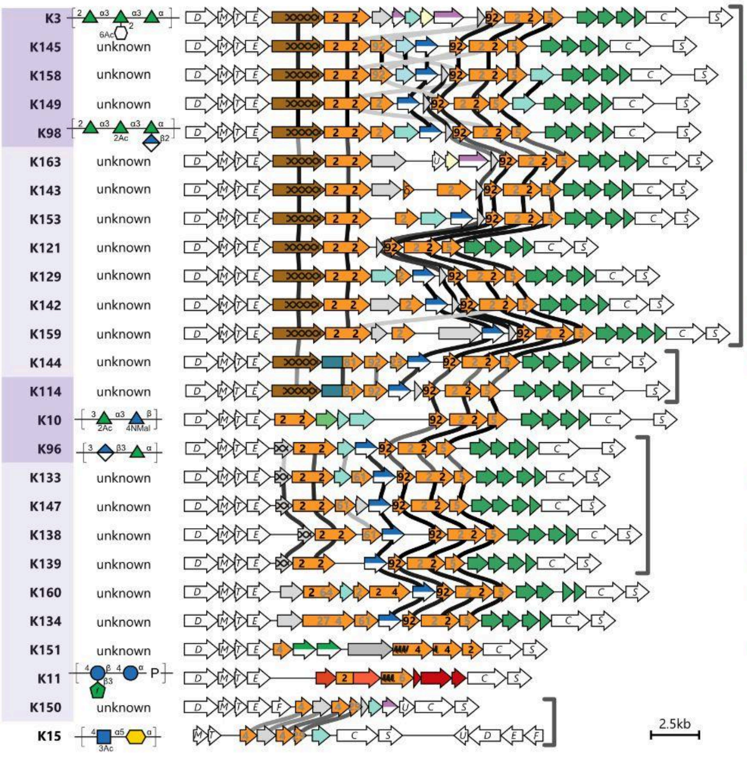

	Locus (Type)	Confidence (%ID, %COV)
K3	KL3 (K3)	Typeable (100.0%, 100.0%)
K145	KL147 (KL147)	Typeable (100.0%, 99.7%)
K158	KL147 (KL147)	Typeable (98.2%, 99.6%)
K149	KL143 (KL143)	Typeable (98.9%, 100.0%)
K98	KL98 (K98)	Typeable (100.0%, 100.0%)
K163	KL140 (KL140)	Typeable (97.2%, 86.2%)
K143	KL166 (KL166)	Typeable (99.9%, 100.0%)
K153	KL152 (KL152)	Untypeable (95.7%, 95.3%)
K121	KL140 (KL140)	Typeable (96.6%, 85.7%)
K129	KL140 (KL140)	Typeable (100.0%, 100.0%)
K142	KL140 (KL140)	Typeable (96.4%, 90.8%)
K159	KL140 (KL140)	Untypeable (94.5%, 86.0%)
K144	KL152 (KL152)	Typeable (98.2%, 74.1%)
K114	KL150 (KL150)	Typeable (99.9%, 100.0%)
K10	KL10 (K10)	Typeable (99.9%, 100.0%)
K96	KL96 (K96)	Typeable (99.9%, 99.4%)
K133	KL132 (KL132)	Typeable (98.1%, 91.5%)
K147	KL132 (KL132)	Typeable (99.0%, 95.8%)
K138	KL132 (KL132)	Typeable (99.3%, 91.7%)
K139	KL132 (KL132)	Typeable (98.6%, 87.6%)
K160	KL162 (KL162)	Typeable (97.8%, 77.2%)
K134	KL136 (KL136)	Typeable (100.0%, 100.0%)
K151	KL160 (KL160)	Typeable (97.2%, 62.8%)
K11	KL11 (K11)	Typeable (99.7%, 100.0%)
K150	KL151 (KL151)	Typeable (99.7%, 100.0%)
K15	KL15 (K15)	Typeable (100.0%, 100.0%)

Supplementary Figure 1

Supplementary Figure 2. Comparison of disrupted/degraded *kps* loci (assigned as unique KLs in EC-K-typing DB) with the corresponding reference clusters in EC-K-typing DB and kTYPr. Each panel shows a parental *kps* locus with repeat unit structure (where available), functional annotations (as depicted in Fig. 2) and a clinker comparison of I. the reference cluster from the kTYPr database, (ii) the equivalent reference cluster from EC-K-typing DB, and (iii) reference KLs from EC-K-typing DB (v6.11.2025) that are disrupted/degraded versions of the parental locus (Supplementary Table 8). Gene colours in the clinker comparison are automatically assigned. To the right of each EC-K-typing DB locus (under "kTYPr result") is the kTYPr evaluation of that locus (Supplementary Table 9). The disrupted/degraded *kps* loci depicted are KL130 (A), KL174 (B), KL170 (C), KL175, KL148, KL156 (D), KL127, KL153 (E), KL155, KL145, KL171 (F), KL164 (G), KL114 (H), KL169 (I), KL149, KL159, KL157 (J), KL138 (K), KL128 (L), KL173 (M), KL158 (N).

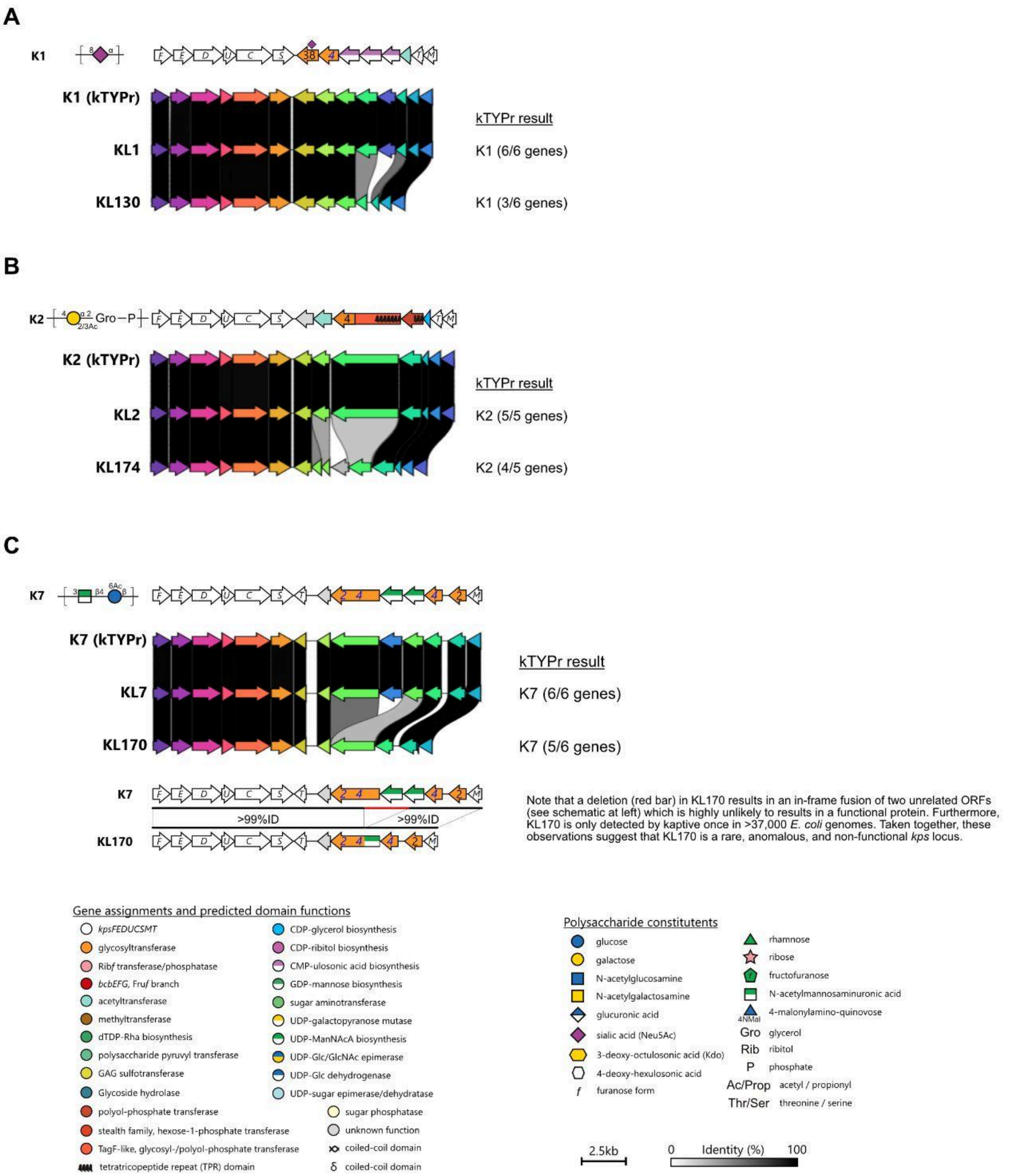

Supplementary Figure 2

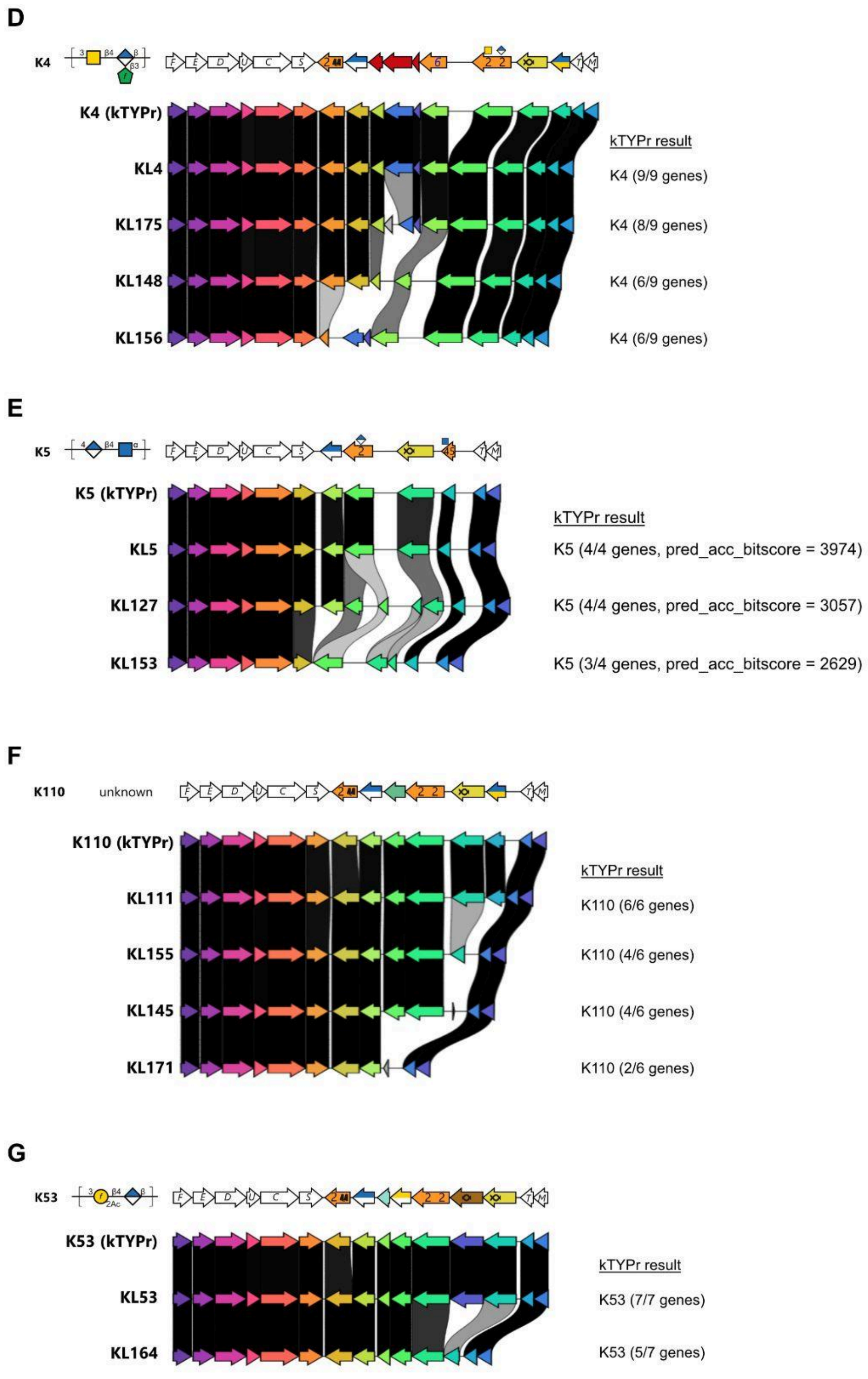

Supplementary Figure 2

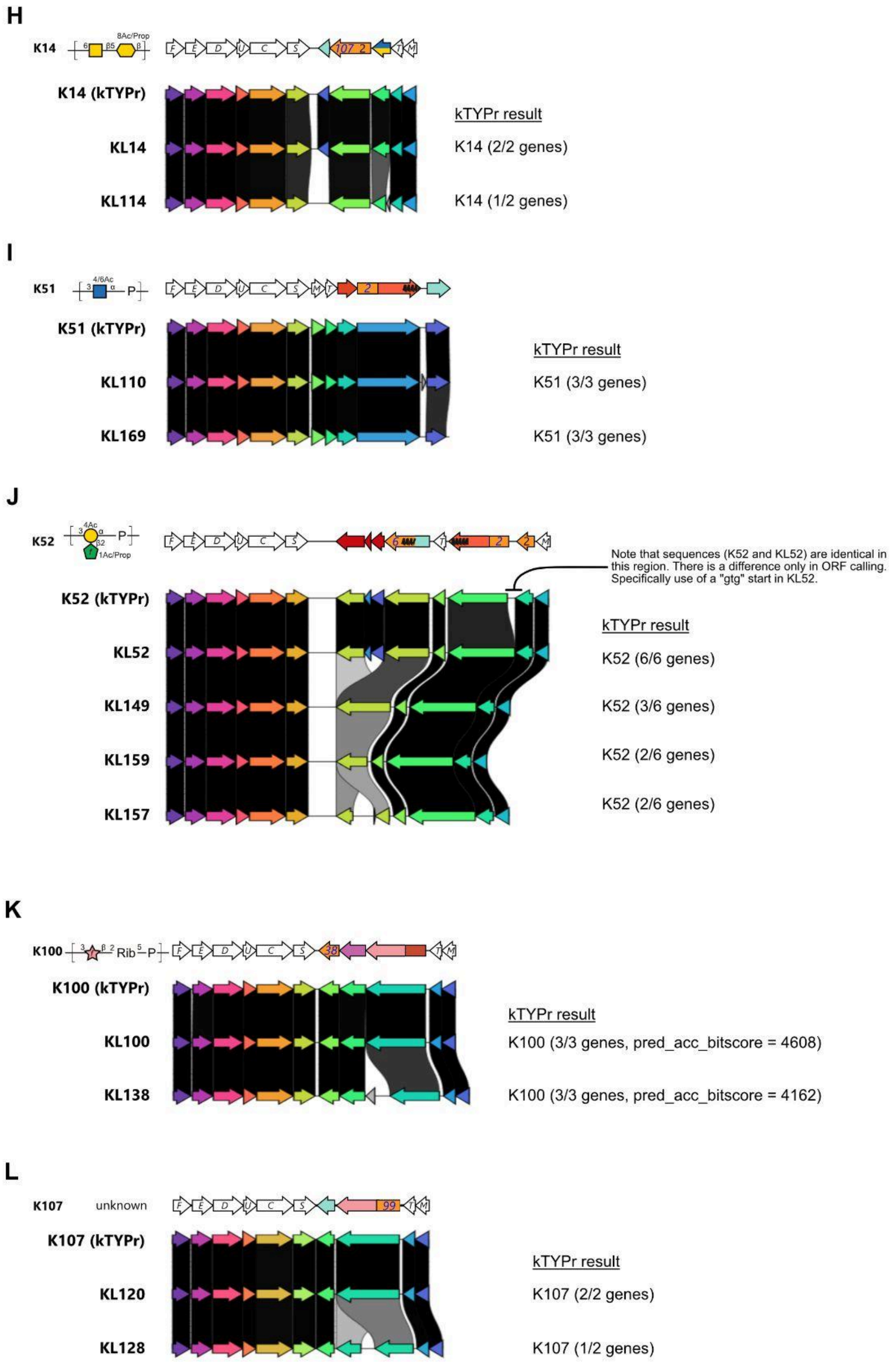

Supplementary Figure 2

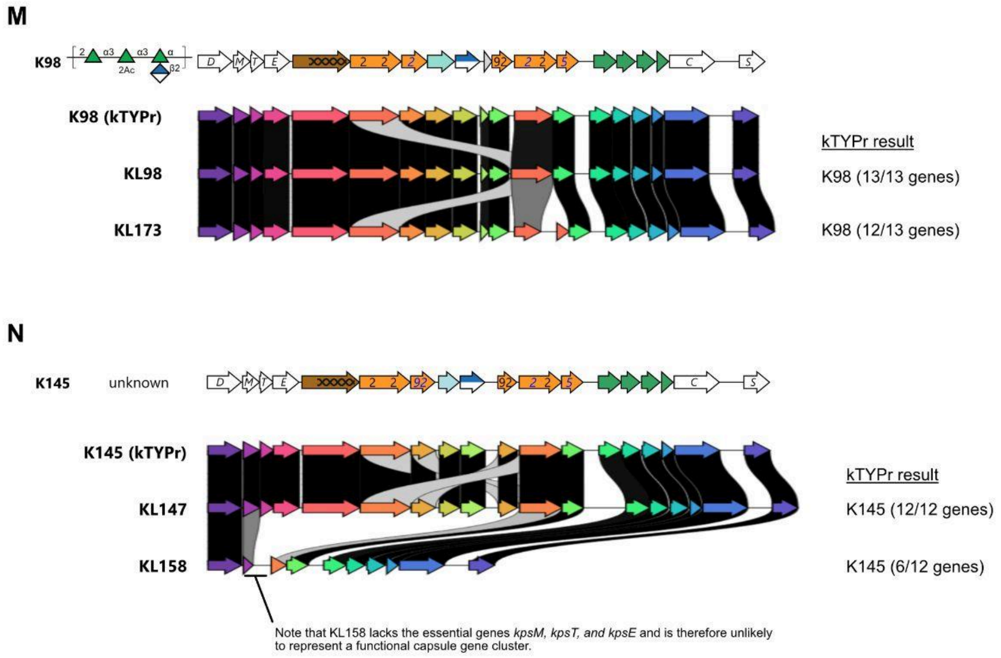

Supplementary Figure 2

Supplementary Tables

Supplementary Table 1. K antigen reference strains used in this study. This table reports the source of each serotype reference strain, the assignment by kTYPr in case differing from the established name, the genome accession, the reported capsule biosynthesis type according to Kunduru and colleagues (https://www.iith.ac.in/EK3D/)[3], references for the structures presented in **Fig. 2**, and the presence (Y; empty otherwise) of conserved genes (*kpsFEDUCSMT*).

Supplementary Table 2. Summary of reference clusters for each K-type in the kTYPr catalog, including K-type, lineage, reference genome accession, RefSeq complete accession (including version), record locus accession corresponding to the record presenting the *kps* cluster, and the genomic coordinates of the *kps* cluster as start..end.

Supplementary Table 3. Identification of conserved domains in serotype-specific ORFs according to Interproscan (Methods).

Supplementary Table 4. Identification of CAZy domains in serotype-specific ORFs according to dbCAN3 (Methods).

Supplementary Table 5. Clustering of serotype-specific ORFs according to sequence similarity (sheet: MMseqs2_all_v_all) and structural similarity (sheet: Foldseek_all_v_all) and identification of structural homologs in the PDB (sheet: Foldseek_all_v_pdb) (Methods).

Supplementary Table 6. Pairwise nucleotide and amino acid identity between all genes and proteins within analyzed *kps* clusters. BLASTP and BLASTN are shown after merging classic output format 6 by the query and subject sequence identifiers (qseqid, sseqid). Then, preceded by prefixes 'BLASTP_' or 'BLASTN_', we show percentage identity (pident), alignment length, number of mismatches and gap openings (mismatch, gapopen), start and end positions in the query and subject (qstart, qend, sstart, send), and the statistical significance and score of the match (evalue, bitscore). Gene/protein identifiers were defined as K-type__Gene (e.g., K2__kpsM corresponds to kpsM from K2). Note that for many cases conservation is only present at amino acid level, thus BLASTN fields appear empty/not_assigned.

Supplementary Table 7. Equivalent types in kTYPr and EC-K-typing DB (v06.11.2025)[2].

Supplementary Table 8. Summary of kTYPr results on EC-K-typing DB (v06.11.2025)[2].

Supplementary Table 9. Results of kTYPr analysis of reference loci in EC-K-typing DB (v06.11.2025)[2].

Supplementary Table 10. Results of Kaptive analysis using EC-K-typing DB (v06.11.20250)[2] on kTYPr reference genomes.

Supplementary Table 11. Results of Kaptive analysis (EC-K-typing DB v6.11.2025)[2] on 37,723 RefSeq genomes.

Supplementary Table 12. Metadata-annotated and K-type profiled collection of 827 genomes from a published study on urosepsis[4] (Methods).

Supplementary Table 13. Metadata-annotated, dereplicated and K-typed collection of 23,188 genomes from NCBI (Methods).

Supplementary Table 14. Metadata-annotated and K-typed collection of 2,762 *E. coli* MAGs from 25 published studies.

Supplementary Table 15. Odds ratios, standard errors, 95% confidence intervals, and p-values obtained from a two-sided Wald z-test for the contribution of each K-type to invasiveness according to a multivariable logistic regression model (Methods).

Supplementary Table 16. Common genes flanking *kps* clusters.

Supplementary Table 17. HMM-specific cutoffs presented as HMM_ID in the first column and the cutoff employed by kTYPr in the second.

Supplementary Table 18. Perturbation analysis. We report the locus tag of the perturbed gene (gene_id), the input sequence identifier (query), the type of artificial modification applied (modification_type; identity mutation, N-terminal trim, C-terminal trim, or dual trim) and its magnitude (value), the HMM profile tested (subject), the resulting HMM alignment score (bitscore) and significance (evalue), the internal kTYPr threshold for that HMM (HMM_cutoff), and whether the hit passed the cutoff (pass_cutoff, 1 if bitscore ≥ cutoff, 0 otherwise).

Supplementary Table 19. Perturbation analysis summary. For each HMM profile (HMM_id), we relate to the minimum sequence identity tolerated while still passing kTYPr cutoffs (min_identity), the maximum tolerated N-terminal truncation (max_N_trimming), the maximum tolerated C-terminal truncation (max_C_trimming), and the maximum simultaneous N- and C-terminal truncation per terminus (max_NC_trimming, e.g. 20 corresponds to 20% trimming at each terminus, 40% total).

References

1. Lam, M. M. C., Wick, R. R., Judd, L. M., Holt, K. E. & Wyres, K. L. Kaptive 2.0: updated capsule and lipopolysaccharide locus typing for the Klebsiella pneumoniae species complex. *Microb. Genom.* **8**, (2022).
2. Gladstone, R. A. *et al.* Group 2 and 3 ABC-transporter-dependent capsular K-loci contribute significantly to variation in the estimated invasive potential of *Escherichia coli*. *medRxiv* (2024) doi:10.1101/2024.11.22.24317484.
3. Kunduru, B. R., Nair, S. A. & Rathinavelan, T. EK3D: anE.coli K antigen 3-dimensional structure database. *Nucleic Acids Res* **44**, D675–D681 (2016).
4. Cuénod, A. *et al.* Bacterial genome-wide association study substantiates papGII of Escherichia coli as a major risk factor for urosepsis. *Genome Med.* **15**, (2023).
